# An evaluation of copy number variation detection tools for cancer using whole exome sequencing data

**DOI:** 10.1186/s12859-017-1705-x

**Published:** 2017-05-31

**Authors:** Fatima Zare, Michelle Dow, Nicholas Monteleone, Abdelrahman Hosny, Sheida Nabavi

**Affiliations:** 1 Computer Science and Engineering Department, University of Connecticut, Storrs, CT USA; 2 Biomedical Informatics Department, University of California San Diego, San Diego, CA USA; 3 Computer Science and Engineering Department and Institute for Systems Genomics, University of Connecticut, Storrs, CT USA

**Keywords:** Copy number variation, Whole-exome sequencing, Somatic aberrations, Cancer

## Abstract

**Background:**

Recently copy number variation (CNV) has gained considerable interest as a type of genomic/genetic variation that plays an important role in disease susceptibility. Advances in sequencing technology have created an opportunity for detecting CNVs more accurately. Recently whole exome sequencing (WES) has become primary strategy for sequencing patient samples and study their genomics aberrations. However, compared to whole genome sequencing, WES introduces more biases and noise that make CNV detection very challenging. Additionally, tumors’ complexity makes the detection of cancer specific CNVs even more difficult. Although many CNV detection tools have been developed since introducing NGS data, there are few tools for somatic CNV detection for WES data in cancer.

**Results:**

In this study, we evaluated the performance of the most recent and commonly used CNV detection tools for WES data in cancer to address their limitations and provide guidelines for developing new ones. We focused on the tools that have been designed or have the ability to detect cancer somatic aberrations. We compared the performance of the tools in terms of sensitivity and false discovery rate (FDR) using real data and simulated data. Comparative analysis of the results of the tools showed that there is a low consensus among the tools in calling CNVs. Using real data, tools show moderate sensitivity (~50% - ~80%), fair specificity (~70% - ~94%) and poor FDRs (~27% - ~60%). Also, using simulated data we observed that increasing the coverage more than 10× in exonic regions does not improve the detection power of the tools significantly.

**Conclusions:**

The limited performance of the current CNV detection tools for WES data in cancer indicates the need for developing more efficient and precise CNV detection methods. Due to the complexity of tumors and high level of noise and biases in WES data, employing advanced novel segmentation, normalization and de-noising techniques that are designed specifically for cancer data is necessary. Also, CNV detection development suffers from the lack of a gold standard for performance evaluation. Finally, developing tools with user-friendly user interfaces and visualization features can enhance CNV studies for a broader range of users.

**Electronic supplementary material:**

The online version of this article (doi:10.1186/s12859-017-1705-x) contains supplementary material, which is available to authorized users.

## Background

Recently, biomedical researchers have considered the impact of genomics variations on human diseases as it provides valuable insight into functional elements and disease-causing regulatory variants [1–3]. Specific focus is drawn on copy number variation (CNV), which is a form of structural variation of the DNA sequence, including multiplication and deletions of a particular segment of DNA (> 1 kb) [4]. The interest and importance of CNVs has risen in a wide collection of diseases including Parkinson [5], Hirschsprung [6], diabetes mellitus [7], Autism [8–10], Alzheimer [11], schizophrenia [12] and cancer [13]. Specifically, significant effort has found associations between CNVs and cancers [13–16]. Cancer is well known as a disease of genome and genomic aberrations of interest in cancer are mostly somatic aberrations, since tumors arise from normal cells with acquired aberrations in their genomic materials [16, 17]. CNV is one of the most important somatic aberrations in cancer [13, 17–19], since oncogene activation is often attributed to chromosomal copy number amplification, and tumor suppressor gene inactivation is often caused by either heterozygous deletion associated with mutation or by homozygous deletion. Thus identification of somatic CNV can have an important role in cancer prognosis and treatment improvement [20].

Array-based technologies have been used widely since late 1990s for more than a decade as an affordable and relatively high-resolution assay for CNV detection [21]. However, array-based technologies have limitations associated with hybridization, which results in poor sensitivity and precision; and with resolution, related to the coverage and density of the array’s probes. With the arrival of next generation sequencing (NGS) technologies [22], sequence-based CNV detection has rapidly emerged as a viable option to identify CNVs with higher resolution and accuracy [14, 23, 24]. As a result, recently whole-genome sequencing (WGS) and whole-exome sequencing (WES) have become primary strategies for NGS technologies in CNV detection and for studying of human diseases. In most cases, CNVs are identified from WGS data. Yet, WGS is considered too expensive for research involving large cohort and WES, which is targeted to protein coding regions (less than 2% of the genome), is becoming an alternative, cost-effective strategy [25]. Even though WES has several technical issues [26], it has been emerged as one of the most popular techniques for identifying clinically relevant aberrations in cancer [27]. WES, can offer lower cost, higher coverage, and less complex data analysis, which are appealing for clinical application when there are several samples. Exome represents a highly function-enriched subset of the human genome, and CNVs in exome are more likely to be disease-causing aberrations than those in nongenic regions [28, 29].

Many tools have been developed for CNV detection using WGS data. However, these methods are not suitable for WES data since their main assumptions on read distributions and continuity of data do not hold. In addition, WES data introduce biases due to hybridization, which do not exist in WGS data and are not considered in the CNV detection methods. On the other hand, germline and somatic CNVs are very different in their overall coverage of the genome and their frequency across population; and they need to be identified differently. The characteristics of somatic CNVs need special consideration in algorithms and strategies in which germline CNV detection programs are usually not suited for. In general, germline CNVs cover small portion of the genome (about 4%) [30], they are more deletion, and they are common among different people. However somatic CNVs can cover a majority part of a genome, can be focal, and are unique for each tumor. As a result CNV detection methods that are developed for identifying population CNVs or germline CNVs cannot be used for identifying somatic aberrations. Also, identifying somatic CNVs in cancer is very challenging because of the tumor heterogeneity and complexity: tumor samples are contaminated by normal tissue, the ploidy of tumors is unknown, and there are multiple clones in tumor samples. On top of the tumor samples’ complexity there are experimental, technical and sequencing noise and biases which makes somatic CNV detection very challenging.

Even though many CNV detection tools and methods have been developed since introducing NGS data, there are few tools available for somatic CNV detection for WES data in cancer. Because of the popularity of WES in cancer studies and challenges of detecting somatic CNV using WES data, in this study we focus on CNV detection methods and tools for WES data in cancer. The objectives of this study are addressing the limitations of the current tools and methods and providing guidelines for developing new ones. In this work first, we briefly explain the CNV detection methods and challenges for WES data and then introduce the recent CNV detection tools for WES data. Then we present the performance analysis of the tools in terms of sensitivity and specificity of detecting true CNVs, using real data and simulated data.

## Methods

### CNV detection methods

In general there are three main approaches to identify CNV from next generation sequencing data: 1) read count, 2) paired-end, 3) assembly [31]. In the read depth (RD) approach mostly a non-overlapping sliding window is used to count the number of short reads that are mapped to a genomic region overlapped with the window. Then these read count values are used to identify CNV regions. Due to reducing the cost of sequencing and improving the sequencing technologies more and more high-coverage NGS data are available; as a result, RD-based methods have recently become a major approach to identify CNVs. Paired-end (PE) approach, which are applied to paired-end NGS data, identifies genomics aberration based on the distances between the paired reads. In paired-end sequencing data, reads from the two ends of the genomics segments are available. The distance between a pair of paired-end reads is used as an indicator of a genomics aberration including CNV. A genomic aberration is detected when the distance is significantly different from the predetermined average insert size. This approach is mostly used for identifying other type of structural variation (beyond CNVs) such as inversion and translocation. In the assembly approach short reads are used to assemble the genomics regions by connecting overlapping short reads (contigs). CNV regions are detected by comparing the assembled contigs to the reference genome. In this methods short reads are not aligned to the reference genome first. Since in WES targeted regions are exonic regions, they are very short and discontinuous across the genome. As a result, the PE and assembly approaches for identifying CNVs are not suitable for WES data. Also high coverage of WES data makes the RD approach more practical. Therefore, all CNV detection tools for WES are based on the RD approach.

In general, the RD approach consist of two major steps: 1) preprocessing, and 2) segmentation. The input data are aligned short reads in BAM, SAM or Pileup formats. In the preprocessing step, WES data’s biases and noise are eliminated or reduced. Normalization and de-noising algorithms are the main components of this step. In the segmentation step a statistical approach is used to merge the regions with the similar read count to estimate a CNV segment. The most commonly used statistical methods for segmentation are circular binary segmentation (CBS) and hidden Markov model (HMM). In CBS, the algorithm recursively localizes the breakpoints by changing genomic positions until the chromosomes are divided into segments with equal copy numbers that are significantly different from the copy numbers from their adjacent genomic regions. In HMM the read count windows are sequentially binned along the chromosome according to whether they are likely to measure an amplification, a deletion, or a region in which no copy number change occurred. Even though other statistical methods have been introduced for detecting CNVs from WGS data, these two methods are the most common methods that are used in the current CNV detection tools for WES data.

### Challenges for detecting somatic CNVs in cancer

Despite improvements to sequencing technologies and CNV detection methods, identifying CNV is still a challenging problem. Complexity of tumors and technical problems of WES add more challenges to identifying somatic CNVs from WES data in cancer [31, 32]. In this section we briefly explain the challenges that somatic CNV identification are faced with in cancer when using WES data. We divide these challenges into three classes: challenges due to 1) sequencing data, 2) WES technical problems, and 3) tumor complexity.

#### Challenges due to sequencing data

The main assumption of the RD based CNV detection algorithms is that the read counts and CNV for a particular region are correlated. However, there are biases and noise that distort the relationship between the read count and copy number. These biases and noise include GC bias, mappability bias, experimental noise, and technical (sequencing) noise. GC content varies significantly along the genome and has been found to influence read coverage on most sequencing platforms [33, 34]. In the alignment step, a huge number of reads are mapped to multiple positions due to the short read length and the presence of repetitive regions in the reference genome [34, 35]. These ambiguities in alignment can produce unavoidable biases and error in RD based CNV detection methods [33]. Furthermore, sample preparation, library preparation and sequencing process introduce experimental and systematic noise that can hinder CNV detection [34, 36].

#### Challenges due to WES technical problem

The exome capture procedure in the library preparation process for WES introduces biases and noise that distorts the relation between read count and CNV. In the WES library preparation, the hybridization process produces biases. In addition, the distribution of read in the exonic regions is not even, which is another source of bias [37]. It is very common that in some genomic regions the read count is very low. This low read counts affect the statistical analysis for calling CNVs and as a result produce noise in the CNV detection algorithms.

#### Challenges due to tumor complexity

Complexity of cancer tumor also distorts the relationship between read count and CNV and as a result produces noise. The tumor complexity includes tumor purity, tumor ploidy, and tumor subclonal heterogeneity. Tumor samples are mostly contaminated by normal cells. Therefore, mapped read on a particular region are not all belong to tumor cells. As a result, read count values do not completely reflect copy number of tumor cells and the tumor normal copy number ratio is less than the real value. This introduces difficulties in calling copy number segments. A threshold for calling CNV will depend on tumor purity, which is usually unknown. There are a few tools available to estimate tumor purity [38, 39]. Aneuploidy of the tumor genome is observed in almost all cancer tumors [40], which creates difficulties in determining the copy number values. The normal tumor read count ratio is corresponding to the average ploidy, which is usually unknown in the tumor sample. It is observed that multiple clonal subpopulations of cells are present in tumors [41]. Due to their low percentage in a sample, it is hard to determine the subclones. This intra-tumor heterogeneity or multiple clonality distorts the CNV and makes calling CNV segments complicated.

### CNV detection tools

AS of August 2016, we have identified fifteen sequence-based CNV detection tools (Additional file 1: Table S1) for WES data. Several studies have already evaluated and compared the performance of CNV detection tools for WES data [31, 32, 42]. However, the focus of their work has not been on cancer. In this work, we restricted the analysis and comparison of CNV tools to those that have been used or have the ability to detect cancer specific aberrations (somatic aberrations). Due to the fast advancing sequencing technologies, we also focused on the widely used and more recent tools. Out of the available CNV detection tools for WES data, we chose the tools that fit the criteria of (1) ability to detect somatic aberration, (2) using read depth (RD) method and (3) was published in the recent years or commonly used. Six tools meet the above criteria: (1) ADTEx [25], (2) CONTRA [43], (3) cn.MOPS [44], (4) ExomeCNV [45], (5) VarScan2 [46], and (6) CoNVEX [47]. ADTEx and CoNVEX were developed by the same group using a similar method, which ADTEx is the modified version of the CoNVEX. As a result, we only considered ADTEx. More recent tools, such as CANOES [48], ExomDepth [49], and cnvCapSeq [50], are not used specifically for cancer; therefore we did not consider them in this study. The list of the tools that we considered in this study and their general characteristics are provided in Table 1.

**Table 1:** Selected tools for the performance analysis of CNV detection tools using WES data

Tool name	ADTEx	CONTRA	cn.MOPS	ExomeCNV	VarScan 2
Chara- Cteristics					
Control set required	Yes	Yes	No	Yes	No
Prog. Language	Python, S/R	Python, R	R	R	Java
Input format	BAM, BED	BAM, SAM, BED	BAM, Read count matrices	BAM, Pileup, GTF	BAM, Pileup
Segmentation Algorithm	HMM	CBS	CBS	CBS	NA^a^
OS	GNU, Linux	Linux, Mac OS	Linux, Mac OS, windows	Linux, Mac OS, windows	Linux, Mac OS, windows
Methodology characteristic	DWT^c^ for de-noising, use BAF^d^	Base-level log-ratio	Bayesian approach for de-noising	Statistical test for analyzing BAF data	CMDS^b^ for generating read counts
Year	2014	2012	2012	2011	2012
URL	http://adtex.sourceforge.net	https://sourceforge.net/projects/contra-cnv/	http://www.bioinf.jku.at/software/cnmops/cnmops.html	https://secure.genome.ucla.edu/index.php/ExomeCNV_User_Guide	http://varscan.sourceforge.net/

^a^Segmentation is not imbedded in the tool. CBS is recommended for segmentation

^b^Correlation Matrix Diagonal Segmentation

^c^Discrete wavelet transform

^d^B allele frequencies

ADTEx [25] is specifically designed to infer copy number and genotypes using WES from paired tumor/normal samples. ADTEx uses both read count ratios and B allele frequencies (BAF) to detect CNV along with their genotypes. It addresses the problem of tumor complexity by employing BAF data, if these data are available. For normalization, ADTEx first calculates the average read count of exonic regions for both tumor and normal, and then computes the ratios of read counts for each exonic region. ADTEx also uses the Discrete Wavelet Transform approach as a preprocessing step to reduce the noise of read count ratio data. It uses the HMM method for segmentation and CNV call. Two HMMs are used in the detection algorithm: one to detect CNVs in combination with BAF signal to estimate the ploidy of the tumor and predict the absolute copy numbers, the other to predict the zygosity or genotype of each CNV segment. When the BAFs of tumor samples are available, they fitted the HMM for different base ploidy values. To determine the base ploidy, ADTEx selects the SNPs which overlaps with each exonic region, segments BAFs using CBS algorithm, estimates B allele count for different ploidy levels, and finally uses the distances between B allele counts to provide the best fit for base ploidy.

CONTRA [45] is a method used for CNV detection for targeted resequencing data, including WES data. It is designed to detect CNV for very small target regions ranging between 100 to 200 bp. The main difference between CONTRA and the other method is that it calculates and normalizes the read count and log ratio for each base (not a window or exon). This allows for better GC normalization and log ratio calculations for low coverage regions. After calculating base-level log ratios, it estimates region-level log ratios by averaging the base-level log ratios over the targeted regions (exons in WES). Then, it normalizes the region-level log ratios for the library size of control and normal samples. The significant values of the normalized region-level log ratios are calculated by modeling region-level log ratios as normal distribution. For detecting large CNVs spanning multiple targeted regions (exons), CONTRA performs CBS on region-level log-ratios. To call a CNV segment, at least half of the segment has to have overlap with the significant region-level CNVs. This method addresses the problems of some very low coverage regions and sequencing biases (GC bias), which are due to uneven distribution of reads in WES.

The main difference between cn.MOPS [44] and other tools is that it can use several samples for each genomics region to have a better estimate of variations and true copy numbers. cn.MOPS uses non-overlapping sliding window to compute read counts for genomic regions. To model read count, it employs a mixture of Poisson distribution across the samples. The model is used to estimate copy number for each genomic region. cn.MOPS does not calculate ratios of case and control. Instead it uses a metric that measure the distance between the observed data and null hypothesis, which is all samples have copy number of 2. If CNV differs from 2 across the sample, the metric is higher. This metric is used for segmentation by CBS per sample. At each genomic position, cn.MOPS uses the model of read counts across samples, so it is not affected by read count alteration along chromosomes. By using Baysian approach, cn.MOPs can estimate noise and so it can reduce the false discovery rate (FDR).

ExomeCNV is designed specifically for WES data using pairs of case-control samples such as tumor-normal pairs. It counts the overlapping reads for exons; and by using these read counts for tumor and normal, it computes the ratio of read counts for each exonic regions. Hinkley transformation (ratio distribution) is used to infer the normal distribution for the read count ratios. After finding ratios of tumor and normal for exonic regions, CBS is used for segmentation. If the tumor purity is given in advance, ExomeCNV will use it to compute copy numbers. It also can detect loss of heterozygosity (LOH) if BAF data is given. ExomeCNV divides the average read count by the overall exome average read count to normalize the average read count per exon.

VarScan2 [46] is also specifically designed for the detection of somatic CNVs in WES from tumor–normal pairs. To compute the read counts of bases, the algorithm considers only high quality bases (phred base quality ≥20) for tumor and normal samples individually. It does not use a sliding window or exons to generate read count data. Instead, it calculates tumor to normal read count ratios of the high quality bases that full fill the minimum coverage requirement. Then, in each chromosome, consecutive bases that their tumor to normal read count ratios do not change significantly, based on the Fisher’s exact test, are binned together as a genomic region to generate read count data. For each genomic region, copy number alterations are detected and then are normalized based on the amount of input data for each sample. A segmentation algorithm in not embedded into the VarScan2 tool and CBS algorithm is recommended for the segmentation of the genomic regions.

### Data sets

In this work, we used real and simulated WES data to evaluate CNV tools’ performances.

#### Real data

We used ten breast cancer patient tumor-normal pair WES datasets from the cancer genome atlas (TCGA) to evaluate the performance of the CNV detection tools. The list of samples is given in the Additional file 1: Table S2. The WES data were generated by the Illumina Genome Analyzer platform at Washington University Genome Sequencing Center (WUGSC). The aligned BAM files of these 20 samples (10 tumor-normal pairs) were downloaded from The Cancer Genomics Hub (CGHub), https://cghub.ucsc.edu/index.html. We also used array-based CNV data from the same 10 tumor samples as a benchmark for the CNV detection tools evaluation. We downloaded SNP-array level 3 data from the Affymetrix genome-wide SNP6 platform from the TCGA data portal website (https://portal.gdc.cancer.gov/projects/TCGA-BRCA) for the 10 tumors.

#### Simulated data

To evaluate the performance of the tools, we have also used benchmark datasets generated by a CNV simulator, called VarSimLab [51]. VarSimLab is a simulation software tool that is highly optimized to make use of existing short read simulators. Reference genome in FASTA format and sequencing targets (exons in the case of WES) in BED format are inputs of the simulator. A list of CNV regions that are affected by amplifications or deletions is randomly generated according to the simulation parameters. The CNV simulator manipulates the reference genome file and the target file before generating short reads that exhibit CNVs. The output consists of: (i) a list file that contains the synthesized amplifications and deletions in txt format, (ii) short reads with no CNVs as control in FASTQ format, and (iii) short reads with synthesized CNV as case in FASTQ format.

We used VarSimLab to generate simulated short reads of length 100 bp for chromosome 1. We generated synthesized datasets with 3 M, 2 M, 1 M, 0.5 M, 0.1 M, 0.05 M, 0.01 M reads to simulate different coverage values (approximately from 0.2X to 60X in exonic regions). For each coverage value, we generated 10 datasets (70 datasets in total). These simulated data with known CNV regions were used to evaluate the performance of the CNV detection tools in terms of sensitivity and specificity for identifying CNV regions.

### Comparison methods

To evaluate the performance of the tools in terms of sensitivity, false discovery rate (FDR) and specificity for detecting CNVs we compared their detected CNVs with the benchmark CNVs. For this comparison, we utilized two approaches: 1) gene-based comparison, and 2) segment-based comparison. Gene-based comparison analysis indicates the performance of the tools on calling CNVs only on exonic regions, which are the targets of the WES. However, segment-based analysis indicates the performance of the tools on overall calling CNV segments across the genome.

#### Gene-based comparison

For the gene-based comparison, we first annotated the detected CNV segments in the benchmark and samples for both real data and simulated data. We used “cghMCR” R package from Bioconductor [52] to identify CNV genes using Refseq gene identifications. The average of the CNV values of the overlapping CNV segments for each gene is used as the gene CNV value. A threshold of ± *thr* for *log*_*2*_ *ratios* was used for calling CNV genes, that is: amplification for *log*_*2*_ *ratios* > *thr*, deletion for *log*_*2*_ *ratios* < *− thr*, and No CNV for *log*_*2*_ *ratios* between *- thr* and *thr.*

For each tool, we computed sensitivity, specificity and FDR separately for amplification and deletion. If we name the detected CNV value for a specific gene as CNVtest and the benchmark CNV value of the gene as CNVbench, then we can define True Positive (*TP*), False Positive (*FP*), True Negative (*TN*) and False Negative (*FN*) for amplified and deleted genes as given in Table 2.

**Table 2:** Computing *TP*, *FP*, *TN* and *FN* for Gene-Based comparison of the performance of the tools

Amplification	CNVbench > *thr*	CNVbench < *thr*
CNVtest > *thr*	*TP*	*FP*
CNVtest < *thr*	*FN*	*TN*
Deletion	CNVbench < (− *thr*)	CNVbench > (−*thr*)
CNVtest < (−*thr*)	*TP*	*FP*
CNVtest > (−*thr*)	*FN*	*TN*

The sensitivities or true positive rates (TPRs), specificities (SPCs) and FDRs are calculated using the following equations for both amplified and deleted genes.

1 $$ TPR\kern0.5em =\kern0.5em \frac{TP\ }{\left( TP+ FN\right)}, $$

2 $$ FDR=\frac{FP\ }{\left( TP+ FP\right)}, $$

and

3 $$ SPC=\frac{TN\ }{\left( FP+ TN\right)} $$

For each tool we calculated TPRs, SPCs, and FDRs of the tools for all datasets and used their average values.

#### Segment-based comparison

For the segment-based comparison, we focused on comparing the CNV segments between detected CNVs and benchmark CNVs. Similar with the gene-based CNV comparison, we used a threshold (*thr*) to call amplified, deleted and no CNV segments. Comparing CNV regions between detected CNVs and their corresponding benchmark CNVs is more complicated than comparing CNV genes. Detected CNV segments, unlike CNV genes, have different sizes and different start and end positions compared to those of benchmark CNV segments. We used “GenomicRanges” R package from Bioconductor [52] to obtain overlapping regions between detected CNVs and benchmark CNVs. If an amplified/deleted segment of a sample, which has CNV > *thr*/ CNV < −*thr*, has an overlap of 80% or more with a benchmark amplified/deleted segment it was considered as *TP*. If we cannot find an overlap of 80% or more between a detected CNV region and any benchmark CNVs, the detected CNV segment was consider as *FP*. An amplified/deleted segment in the benchmark that does not have an overlap of 80% or more with any detected amplified/deleted regions was called *FN*. Since the regions with no CNVs cover very large sections of a genome we did not calculate *TN* regions. Therefor for segment-based comparison we calculated TPRs and FDRs as eqs. 1 and 2. If we name a CNV segment of samples as *TestSeg* and a CNV segment of benchmark as *BenchSeg*, we can calculate *TPs*, *FPs* and *FNs* as shown in Table 3.

**Table 3:** Computing *TP, FP* and *FN* for Segment-Based comparison

Amplification	BenchSeg CNV *> thr*	BenchSeg CNV *< thr*
TestSeg CNV *> thr*	*TP* if they have overlap >80% of TestSeg	*FP* if they have overlap >80% of TestSeg
TestSeg CNV *< thr*	*FN* if they have overlap >80% of TestSeg	*…*
Deletion	BenchSeg CNV *< − thr*	BenchSeg CNV > *−thr*
TestSeg CNV < −*thr*	*TP* if they have overlap >80% of TestSeg	*FN* if they have overlap >80% of TestSeg
TestSeg CNV > −*thr*	*FN* if they have overlap >80% of TestSeg	*...*

## Results and Discussion

### Real data

#### Gene-based comparison

The average sensitivity, specificity and FDR of the 5 CNV detection tools on real breast cancer WES data are shown in Table 4 (The CNV results of the tools for the real samples are given in Additional files 2, 3, 4, 5 and 6). Thresholds of ±0.2 were used to call CNV genes. In summary tools show moderate sensitivities (~50% to ~80%), fair specificities (~70% to ~94%) and poor FDRs (~30% to 60%) on detecting CNV genes. Of the five tools, ExomeCNV was found to outperform other tools with the highest sensitivity rate of 83.67% for amplification and 81.3% for deletion. VarScan2 (FDR = 26.87%, SPC = 92.71%) and ADTEx (FDR = 41.80%, SPC = 94.18%) show the best FDR and specificity for detecting amplified and deleted genes (Table 4). ExomeCNV employs a minimum power/specificity parameter, and it makes a call on a specific exon if the desired power/specificity is achieved by the coverage of that exon. That is likely the reason of its better performance.

**Table 4:** Overall performance of the CNV detection tools using the gene-based comparison approach for real data

Method	ADTEx	CONTRA	cn.MOPS	ExomeCNV	VarScan2
Amplification					
Sensitivity	51.53%	54.37%	58.03%	**83.67%**	69.11%
FDR	33.70%	53.52%	57.36%	38.79%	**26.87%**
SPC	89.84%	83.06	66.54%	82.07	**92.71%**
Deletion					
Sensitivity	50.14%	64.95%	52.81%	**82.94%**	76.77%
FDR	**41.80%**	64.86%	61.35%	45.31%	51.91%
SPC	**94.18%**	78.86%	78.08%	87.26%	82.52%

In the table, bold value in each line represents the best value of each performance measure

In general, tools show higher FDRs in detecting deleted genes compared to detecting amplified genes. ADTEx, CONTRA, and cn.MOPS show similar rate of sensitivity for detecting the true amplified CNV genes (about 50%). The high FDRs of the tools might be partially due to using array-based CNV results as benchmark CNVs. Array-based technologies suffer from low resolution due to probe intensities, which results in detecting large CNV regions and missing the detection of small CNV regions.

**Fig. 1:**
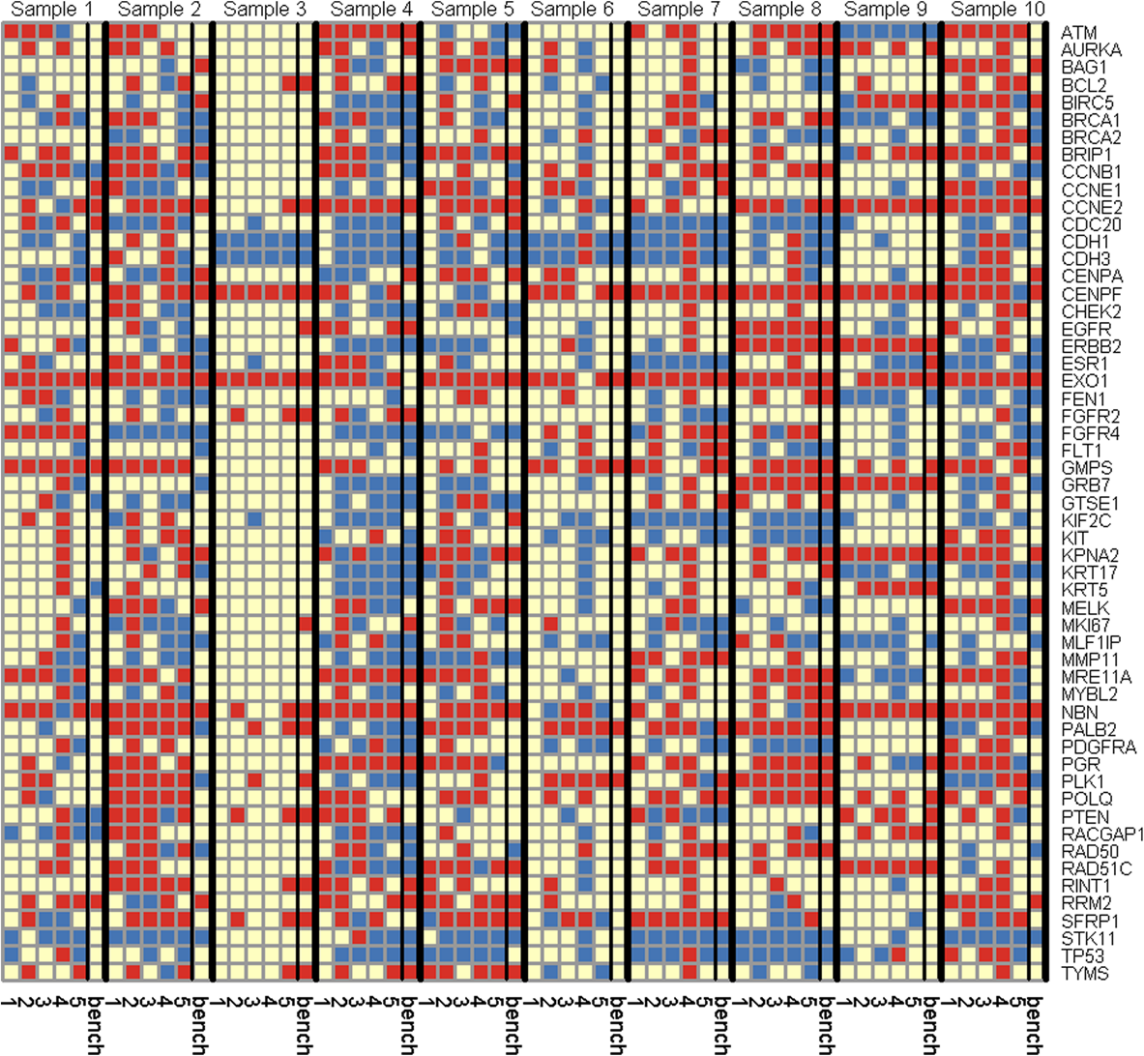
CNV call of 55 breast cancer related genes. *Blue*: deletion, *Red*: amplification, and *light yellow* no CNV call. Order of tools from *left* to *right*: 1: ADTEx, 2: ExomeCNV, 3: CONTRA, 4: cn.MOPS, and 5: VarScan2

**Fig. 2:**
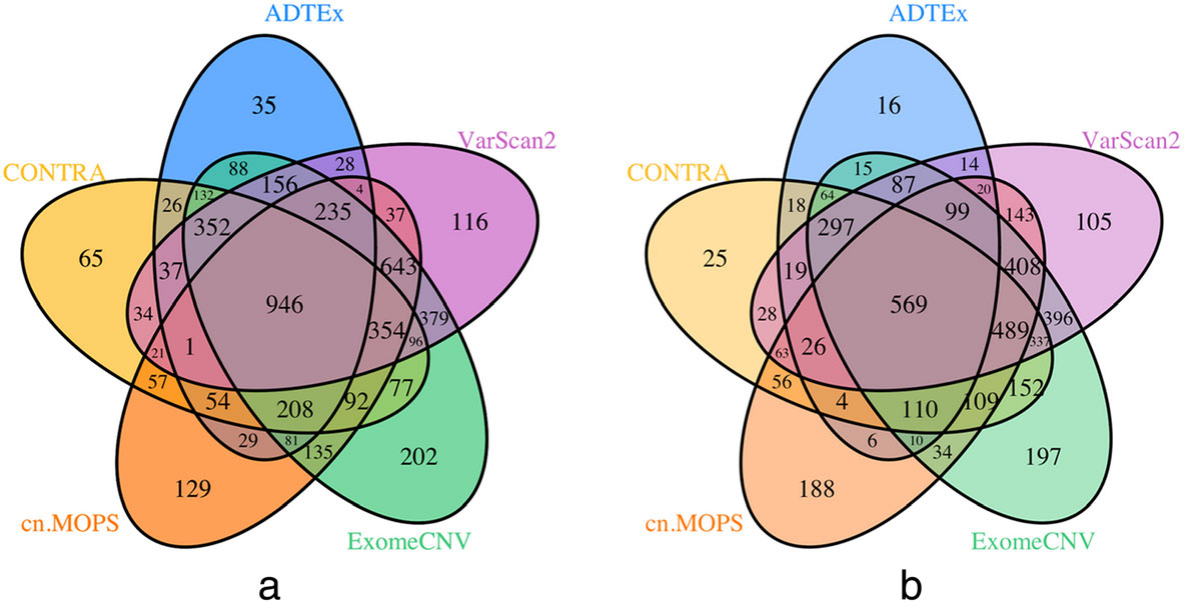
Venn diagrams of the average of the number of truly detected CNV genes from the 5 tools, (**a**) amplified genes, (**b**) deleted genes

To examine the consistency of the tools’ results, we compared the CNV calls of the genes for each sample across the tools. Figure 1 shows the CNV calls of 55 breast cancer related genes [53, 54] for the breast cancer samples used in this study. It can be seen that there is no strong consistency among the tools in calling these breast cancer related genes for each sample. There are few genes that are called as amplified or deleted in each sample by all the tools. Many genes are called as amplified by some tools, deleted by some other tools and no CNV by the rest. As can be seen from Fig. 1, sample 3 has a few amplified or deleted CNV regions compared to other samples; thus, we removed it for the rest of analysis. Figure 2a and b show the Venn diagram of the average of the number of truly detected deleted and amplified genes by the tools from all the samples. As can be seen, a small fraction of true amplified and true deleted genes are common across all the tools. Only 946 genes out of 4849 true amplified genes in union, and 569 genes out of 4104 true deleted genes in union are common across the tools, which show low consistency among the tools.

#### Segment-based comparison

**Fig. 3:**
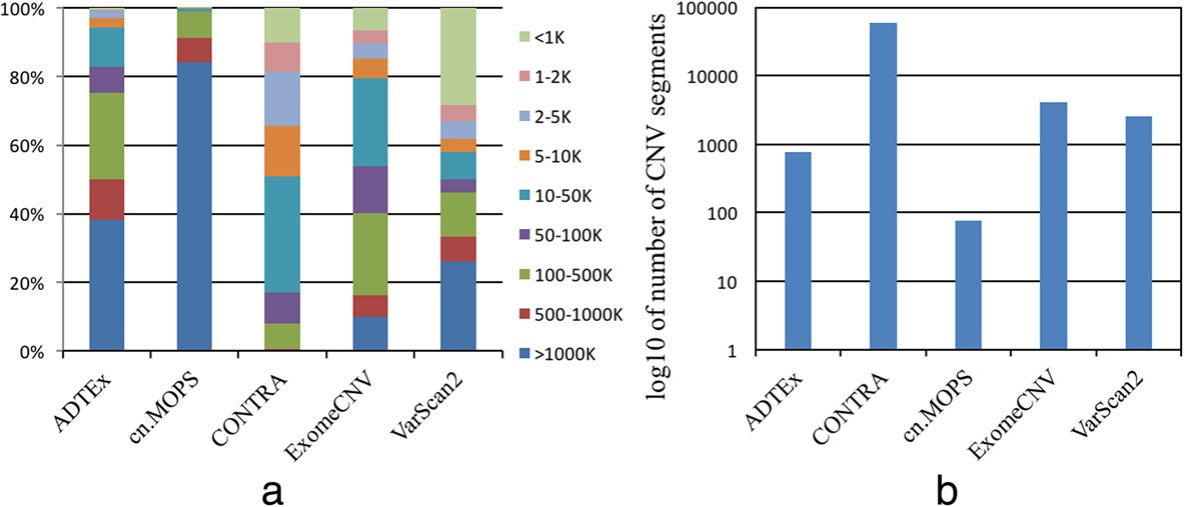
Characteristics of the detected CNV regions by the 5 tools. **a** Size distributions of CNV segments. **b** Number of detected CNV segments

Average sensitivities and FDRs of the CNV detection tools based on the segment-based comparison analysis are given in Table S3 in Additional file 1. We considered an overlap of at least 80% between the detected CNVs and benchmark CNVs to call *TP*s and *FPs*. We also used thresholds of ±0.2 to call CNV regions. Sensitivities and FDRs of the segment-based analysis are almost similar to the sensitivities and FDRs of the gene-based analysis. However, we observed that tools that can detect larger CNV segments show better performance. This is most likely due to use large benchmark CNV regions from the array-based technologies. ExomeCNV and cn.MOPS show the highest sensitivities for detecting CNV Segments; and cn.MOPS and VarScan2 show the lowest FDR for detecting CNV Segments (Additional file 1: Table S3). ExomeCNV and cnMOPS also detect a greater percentage of large CNV segments (Fig. 3a).

The CNV size distributions and the number of the detected CNVs from the breast cancer samples by the five tools are shown in Fig. 3. There is no strong consistency among the tools on the size and number of detected CNVs as well. Tools that detect larger CNV segments detect lower number of CNVs and tools that detect shorter CNV segments detect more CNVs (Fig. 3a and b). That indicates a high level of errors in CNV break point (CNV segment edge) detection. In Fig. 3a, cn.MOPS and ADTEx show a tendency to detect larger CNV segments. CONTERA detects shorter CNV segments. Only about 1% of its detected CNVs regions are larger than 1000 K.

**Fig. 4:**
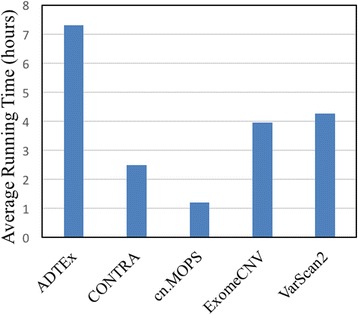
Average execution times of the tools from 5 runs on a real breast cancer dataset

We also examined the computational complexity of these CNV detection tools by comparing their execution times. In order to compare the running time of the tools, we run the tools using one of the breast cancer sample for 5 times and averaged their execution times. The runs performed on a single node of the same computer cluster. Figure 4 shows the average execution times of the tools on the real dataset. In Fig. 4, you can see that while ADTEx takes the longest time, cn.MOPS is the fastest tool among the five tools. The running times of the other three tools are almost comparable.

In summary, ADTEx has a moderate sensitivity and better FDR. Similar to cn.MOPS, it is capable of detecting larger CNV regions, but it detects CNVs with a wider range of sizes. ADTEx is the most recently developed tool for CNV detection. Different from the other four tools, it employs two HMMs for calling CNVS and a denoising method for preprocessing. Its detection method is more computationally expensive compared to the other tools. CONTRA has a moderate sensitivity and FDR, with a wide range of detected CNVs sizes. Its performance outperforms the other tool using simulated data. Because CONTRA was developed based on empirical relationships between log-ratios and read count data, it relies on the case sample being largely copy number neutral. But this might not be true for cancer data, and results in poor performance for real cancer data. cn.MOPS also has a moderate sensitivity and FDR for the gene-based comparison approach. cn.MOPS can apply to multiple samples at once for a better normalization, which can improve its performance. It shows better performance in detecting CNV segments. cn.MOPS detects larger CNV regions, and is the fastest tool. ExomeCNV has higher sensitivity and moderate FDR. Its better sensitivity can be due to its additional step to call CNV at individual exon before segmentation process. In general, ExomeCNV shows better overall performance in comparison to the other tools. Its execution time is comparable with other tools as well. In this study we did not use BAF data. Using BAF data can improve its performance too. VarScan2 has higher sensitivity and better FDR for both amplification and deletion in the gene-based comparison analysis. Even though VarScan2 did not show the best performance, it shows stable overall performance and ease of use with a comparable execution time.

### Simulated data

The advantages of using simulated data are that: 1) we have a known list of benchmark CNVs that can be used as a gold standard for calculating accurate sensitivities and FDRs, and 2) we can investigate the effect of coverage on the detection power of the tools. Since the price of sequencing directly depends on the coverage of the data (or number of reads), knowing the minimum coverage of data needed for accurate CNV detection is important. It is useful to notice that even though simulated data harbor sequencing noise and biases, tumor related distortions have not simulated in the synthesized data. As a result, CNV detection tools show superior performance on synthesized data compared to real tumor data. We generated 7 sets of 10 simulated paired-end WES data for chromosome one. Each set has different numbers of 100 bp reads of 3 M, 2 M, 1 M, 0.5 M, 0.1 M, 0.05 M, 0.01 M. Thresholds of ±0.5 were used to call CNV genes and segments for simulated data.

#### Gene-based approach

**Fig. 5:**
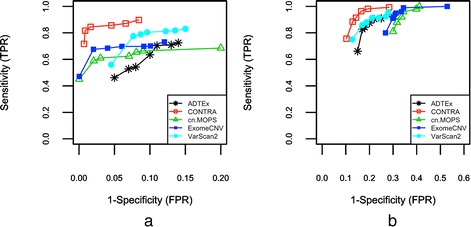
Sensitivity (TPR) versus 1- specificity (FPR) of the tools for different coverage values, using simulated data, for (**a**) amplified genes, and (**b**) deleted genes. Since CONTRA could not generate the proper output for the coverage of 0.01 M, its results for coverage of 0.05 have not been shown

Figure 5a and b show sensitivity (TPR) verses 1- specificity (FPR) of the tools in calling amplified and deleted genes respectively, when changing the number of reads in chromosome 1 from 0.01 M to 3 M. In calling amplified genes, CONTRA was found to outperform other tools with the highest sensitivity rate especially for lower coverage values. Its base-level log2 ratio approach gives it the advantage of working well for low coverage data. In calling deleted CNV genes, the five tools showed comparable performance in terms of sensitivity and FDR. As expected, we can see that the detection power of the tools decreased with lowering the coverage (Fig. 5a and b). We also noticed that the performance of the tools is not improving significantly by increasing the number of read more than about 0.5 M for chromosome 1 (almost the coverage of 10X for the exonic regions).

#### Segment-based approach

Segment-based analysis of the performance of the tools using the simulated data showed that VarScan2 and cn.MOPS have the highest sensitivity for detecting amplified CNVs, and Varscan2 and ExomeCNV have the lowest FDR in detecting deleted CNVs, as shown in the Additional file 1: Table S4. The five tools show almost the same FDR for detecting amplified and deleted CNV segments. They have high sensitivities and low FDRs especially for high coverage values. As expected, we observed that the overall performances of the tools are better for higher coverage values (Additional file 1: Table S4).

In addition, we analyzed False Negative, False Positive and True Positive CNV segments regarding their lengths. We observed that in general FN and FP segments have significantly shorter lengths compared to TP segments (with *p*-value <0.05 using Wilcoxon test) for all of the tools. The boxplots of the lengths of FP, FN, and TP CNV segments for all the tools and for amplification and deletion are given in Additional file 1: Figures S1 and S2). It can be concluded that the power of all the tools in detecting short CNVs is low and they detect many false short CNVs and miss many true ones. The length of a CNV segments is indirectly related to the local coverage of the segment. Therefore, mis-detection of short and low coverage segments is one of the major reasons for poor performance of the tools.

## Conclusions

In this study, we surveyed CNV detection tools for WES data in cancer. We focused on CNV detection for WES data because WES is a more affordable and a more popular sequencing technique in translational research compared to WGS. Despite the popularity and prevalence of WES data, detecting CNV using WES data is challenging. CNV detection using WES data requires different approaches compared to the CNV detection using WGS data due to different type of noise and biases and sparsity of exonic regions. Also, in this study we concentrated our efforts on studying CNV detection tools that can apply to or designed for cancer. Cancer tumors harbor somatic aberrations and tumors are complex due to tumor ploidy, normal cells contamination and subclonal heterogeneity. As a result, studying CNVs in cancer requires different approaches compared to studying germline CNVs or population CNVs.

We evaluated the performance of the five most recent and commonly used CNV detection tools (Table 1) for cancer WES data in terms of sensitivity, FDR and specificity of detecting CNV genes and CNV segments. For the performance evaluation, we used real breast cancer data as well as simulated data. The comparative analysis of the performance of the tools on real data shows that the tools have moderate detection power (sensitivity) while show low precision (or poor FDR). The poor FDRs show that the tools generate many false positives.

There are some important reasons for having low sensitivity and specificity of CNV detection tools. First reason is related to the inability of accurate detection of CNV breakpoints for WES date. Percentage of exons in genome is about 1.1% to 1.4% and some of the real breakpoints are outside of the captured target regions [55]. Second, all of these tools are based on the RD approach that uses the depth of coverage (read count) information for detecting CNV. This method has low resolution and power in detecting small CNVs due to low values of read count data [42]. Third reason is the lack of appropriate preprocessing methods such as bias removing, de-noising and normalization. It is assumed that there is a shared bias between tumor and normal read count data, which can be removed by calculating the ratio of tumor and normal coverage. But this assumption can lead to potential problems. The noise of a local region is not considered in this assumption. Therefor in computing ratio values of depth of coverage between tumor and normal samples, noise is amplified. It has been shown that CNV detection tools do not perform well for low quality and noisy samples [56], which indicates a need for using more advanced preprocessing and detection (segmentation) methods.

Also, the characteristics of the detected CNV segments (size, number, orientation) are different across the tools that show inconsistency in the segmentation of the CNV regions. In addition, the consensus CNVs across the tools is low which can be the result of the high FDRs as well. Using synthesized data resulted in the better performance of the tools because tumor complexity (ploidy, normal contamination, clonal heterogeneity) has not been simulated on the synthesized data. That shows the importance of considering tumor complexity in CNV detection in cancer.

Even though tumor complexity play an important role on the accurate detection of CNVs, there are only two tools, ADTEx and ExomeCNV, that partially address tumor complexity by employing BAF information – regarding tumor ploidy- in their CNV detection methods. However, tumor subclonal heterogeneity and tumor purity have not been addressed by any tools. Incorporating extra information such as allelic frequency and a model of tumor purity can help to improve true detection of CNVs.

Using the simulated data, we also investigated the effect of coverage on the detection power of the tools. Coverage of sequencing, or the number of reads, is proportional to the cost of sequencing. High coverage is important to call somatic aberration, especially for somatic mutations, but it costs more as well. In this study, we observed that the tools’ performances do not improve significantly by increasing the coverage more than about 10X on exonic regions. Although, the tools use different preprocessing methods, they used HMM and CBS for segmentation of CNV regions, which are adopted from CNV detection for microarray technologies. New segmentation approaches that can effectively use characteristics of WES data in cancer, such as tumor complexity and sparsity of exonic regions, need to be developed. In addition, even though it is well known that read count data suffer from noise there is only one tool, ADTEx, that uses a noise cancellation method- based on the discrete wavelet transform technique - to reduce noise before segmentation. Also, somatic CNV detection methods typically utilize matched normal DNA as a means for identifying true somatic variations from germline variations and reducing background sequencing biases. Developing appropriate and effective noise cancelation and normalization methods is required to detect CNVs more accurately. Utilizing techniques from other fields such as statistical image/signal processing can help to address these challenges.

One of the challenges we faced in this study was usability of the tools. One of the problems is the mismatch of the tools with the newer version of their dependencies. In addition, all of the CNV detection tools are command line based software tools without user-friendly user interfaces. The lack of user-friendly user interface makes the tools’ utilization difficult for researcher with limited expertise in computer systems. Visualization is also very important to study CNVs. Most of the tools offer commands for plotting CVNs with very limited features. However, embedding advanced and user-friendly visualization features to the CNV detection software tool can be very useful.

Finally, the lack of a CNV gold standard to accurately evaluate the performance of the tools is another challenge in developing CNV detection tools. An effort on developing a gold standard for CNV detection can significantly help CNV detection tool development.

In summary, the moderate sensitivities and poor FDRs of the current CNV detection tools for WES data in cancer indicate the need for developing more efficient and precise CNV detection methods. CNV detection tools with user-friendly user interfaces and visualization features can extremely enhance CNV studies. Also, utilizing advanced novel segmentation, normalization and de-noising techniques that are designed specifically for cancer data is necessary.

## Additional files

Additional file 1: Supplementary materials (Table S1-S4, Supplementary Figures). (PDF 351 kb)

Additional file 2: CNV results of ADTEx for the 10 real breast cancer samples. (XLSX 572 kb)

Additional file 3: CNV results of CONTRA for the 10 real breast cancer samples. (XLSX 21170 kb)

Additional file 4: CNV results of cn.MOPS for the 10 real breast cancer samples. (XLSX 68 kb)

Additional file 5: CNV results of ExomeCNV for the 10 real breast cancer samples. (XLSX 3535 kb)

Additional file 6: CNV results of VarScan2 for the 10 real breast cancer samples. (XLSX 999 kb)

## Abbreviations

CNV: Copy Number Variation
TCGA: The Cancer Genome Atlas
WES: Whole Exome Sequencing
WGS: Whole genome Sequencing
FDR: False Discovery Rate
BAF: B Allele Frequencies
SPC: Specificity
HMM: Hidden Markov Model
CBS: Circular Binary Segmentation
TP: True Positive
FN: False Negative
TN: True Negative
FP: False Positive
TPR: True Positive Rate
FPR: False Positive Rate
