# Supplementary material for: An evaluation of copy number variation detection tools for cancer using whole exome sequencing data
Source: BMC Bioinformatics. 2017 May 31;18:286. doi: 10.1186/s12859-017-1705-x (PMC5452530; doi:10.1186/s12859-017-1705-x)
Supplement: Supplementary file 1 — Supplementary materials (Table S1-S4, Supplementary Figures). (PDF 351 kb) [file 12859_2017_1705_MOESM1_ESM.pdf]

Supplementary Tables

Table S1. Recent tools for the comparative analysis of CNVs detection in exome sequence data

Tool name	Cancer-specific (somatic aberration)	Control/ control set required	read-depth/ DOC?	Prog. Language	Input Format	Output Format	Segmentation Algorithm	OS	Methodology Characteristic	YEAR	URL
CONTRA	Yes	Yes	Yes	Python, R	BAM, SAM, BED	VCF tab-delimited	CBS	Linux, Mac OS	Base-level log-ratio	2012	https://sourceforge.net/projects/contra-cnv/
VarScan2	Yes	Yes	Yes	Java	BAM, Pileup	Tab-delimited	NA. it is not imbedded in the tool. CBS is suggested	Linux, Mac OS, windows	CMDS<br>For determining window for read count data	2012	http://varscan.sourceforge.net/
ExomeCNV	Yes	Yes	Yes	R	BAM, Pileup, GTF	TXT, PNG	CBS	Linux, Mac OS, windows	Statistical test (Ftest),<br>Use BAF	2011	https://secure.genome.ucla.edu/index.php/ExomeCNV_User_Guide
cn.MOPS	Yes	No	Yes	R	BAM, Read count matrices	R environment variable	CBS	Linux, Mac OS, windows	Bayesian Approach for denoising	2012	http://www.bioinf.jku.at/software/cnmops/cnmops.html
ADTex	Yes	Yes	Yes	Python, S/R	BAM, BED	Tab-delimited file	HMM	GNU, Linux	DWT<br>denoising,<br>Use BAF	2014	http://adtex.sourceforge.net
CoNVEX	Yes	Yes	Yes	R	BAM	Tab-delimited file	HMM	GNU, Linux	DWT denoising	2013	http://adtex.sourceforge.net
Control-FREEC	Yes	No	Yes	C++, R	SAM,BAM,SAM tools pileup, Eland, BED, soap,	regions of gains, losses and LOH, copy number, and BAF	Median BAF segmentation	Linux , Windows<br>for low version	Lasso, GMM, GC<br>-<br>content normalization	2012	http://bioinfo.curie.fr/projects/freec/

ExomeDepth	No	Yes	Yes	R	Read count matrices	Tab-delimited file	HMM	Unix/Linux, Mac OS, Windows	Base-level log-ratio	2012	https://cran.r-project.org/web/packages/ExomeDepth/index.html
CoNIFER	No	No	Yes	Python	BAM, RPKM	Tab-delimited	HMM	Linux, Mac OS	SVD	2012	http://conifer.sourceforge.net/download.html
XHMM	No	No	No	C++, R	GATK's Depth of coverage file	VCF,<br><br>Tab-delimited	HMM	Linux, Mac OS	PCA, HMM	2012	http://atgu.mgh.harvard.edu/xhmm/download.shtml
CANOES	No	Yes	Yes	R	BAM, BED	Pdf	HMM	Unix/Linux, Mac OS, Windows	PCA	2014	https://omictools.com/canoes-tool
CONDEX	No	Yes	Yes	java	BAM, BED	Tab-delimited	HMM	Unix/Linux	Poisson latent factor model	2011	https://code.google.com/archive/p/condr/downloads
EXCAVATOR2	Yes	Yes	Yes	R, Fortran	BAM, BED	TXT, Pdf, PNG	CBS	Linux, Mac OS	MACS-Based peak calling to identify all regions that are well covered by reads (peaks)	2016	https://sourceforge.net/projects/excavator2tool/
PatternCNV	No	No	Yes	Perl, R	BAM, BED	Wig file	CBS	Linux, Mac OS	QC measures designed to identify outlier samples	2014	https://github.com/topsoil/patternCNV/
cnvCapSeq	No	No	Yes	Java	BAM	TXT	HMM	Linux, Mac OS	Normalization using Singular Value Decomposition	2014	https://sourceforge.net/projects/cnvcapseq/

Table S2. List of breast cancer patient tumor-normal pair WES datasets from the cancer genome atlas (TCGA)

List of Samples
TCGA-BH-A0B3
TCGA-A7-A0CE
TCGA-BH-A0DT
TCGA-BH-A0E0
TCGA-BH-A1FC
TCGA-E2-A1LG
TCGA-E9-A1NH
TCGA-BH-A18R
TCGA-BH-A18U
TCGA-AC-A2BK

Table S3. Overall performance of the CNV detection tools using the segment-based approach using real data.

Method	ADTEx	CONTRA	cn.MOPS	ExomeCNV	VarScan2
Amplification					
Sensitivity	51.68%	48.12%	56.76%	**59.92%**	53.86%
FDR	62.72%	57.38%	**55.38%**	59.09%	59.37%
Deletion					
Sensitivity	52.25%	52%	**58.95%**	53.67%	54.06%
FDR	63.83%	54.1%	54.17%	52.99%	**46.56%**

In the table, bold value in each line represents the best value of each performance measure

Table S4. Overall performance of the CNV detection tools using the segment-based approach using simulated data.

Amplification THR=0.5	ADTEX		CONTRA		cn.MOPS		ExomeCNV		VarScan2	
	SEN	FDR	SEN	FDR	SEN	FDR	SEN	FDR	SEN	FDR
3M	85.49%	29.80%	80.45%	27.10%	90.32%	25.66%	85.60%	27.46%	90.07%	22.68%
2M	84.95%	31.57%	79.43%	32.50%	89.90%	27.68%	83.29%	29.53%	88.72%	24.57%
1M	80.09%	34.57%	79.10%	32.51%	88.70%	29.57%	82.21%	38.68%	82.50%	30.8%
0.5M	81.43%	33.68%	76.10%	35.68%	85.43%	28.80%	81.90%	38.77%	83.33%	33.77%
0.1M	79.90%	39.23%	75.65%	42.54%	80.8%	35.75%	76.43%	42.36%	76.63%	35.99%
0.05M	70.12%	41.22%	63.44%	45.65%	72.34%	41.89%	71.20%	45.87%	70.77%	40.05%
0.01M	61.43%	42.67%	…	…	65.54%	45.44%	62.84%	47.77%	63.55%	41.33%
DELETION THR=(-0.5)	ADTEX		CONTRA		cn.MOPS		ExomeCNV		VarScan2	
	SEN	FDR	SEN	FDR	SEN	FDR	SEN	FDR	SEN	FDR
3M	70.56%	29%	78.90%	31.25%	69.71%	29.91%	86.50%	27.02%	90.02%	24.09%
2M	70.11%	29.79%	77.99%	33.68%	68.34%	31.28%	82.20%	32.41%	88.73%	24.97%
1M	65.12%	29.57%	75.42%	34.71%	69.05%	37.43%	81.27%	31.48%	80.26%	34.95%
0.5M	65.43%	34.57%	77%	34.99%	59.32%	36.72%	81.99%	36.41%	80.06%	34.90%
0.1M	63.23%	42.76%	70.87%	39.65%	61.64%	40.41%	75.50%	39.29%	78.48%	40.25%
0.05M	57.42%	45.93%	60.96%	46.30%	55.33%	44.39%	67.33%	42.58%	70.62%	41.76%
0.01M	51.54%	47.90%	…	…	50.72%	50.23%	59.92%	45.91%	62.33%	42.33%

Supplementary Figures

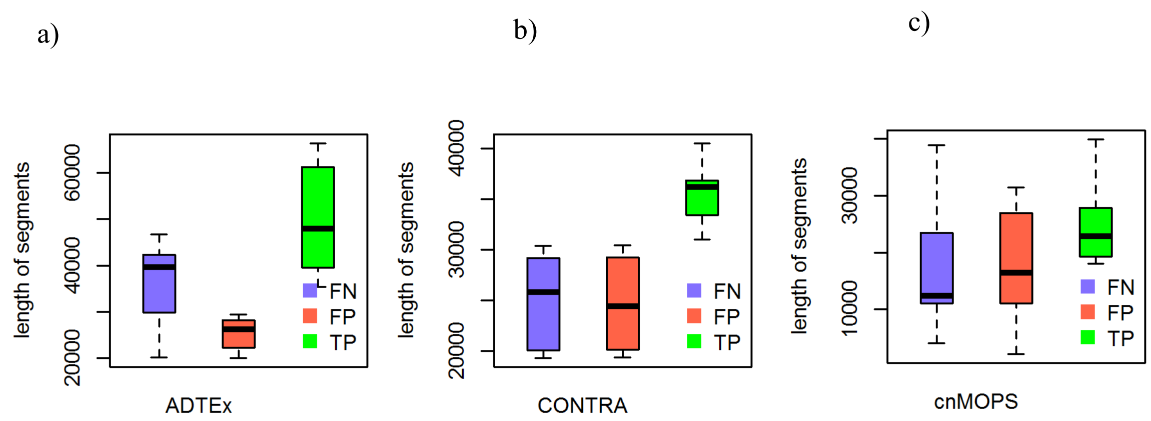

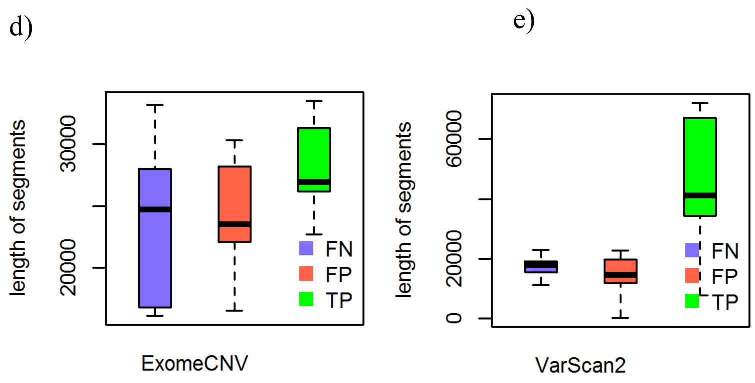

f)

TOOLS	P-Values
ADTEx	0.0452
CONTRA	0.0004
Cn.MOPS	0.0376
ExomeCNV	0.1212
VarScan2	0.0028

Fig. S1. The boxplots of lengths of FP, FN, and TP CNV segments for a) ADTEx, b) CONTRA, c) cn.MOPS, d) ExomeCNV, and e) VarScan2 for amplification . f) P-values from comparing lengths of FN and TP CNV segments using Wilcoxon test. (3M simulated data, Threshold=0.5)

a) b) c)

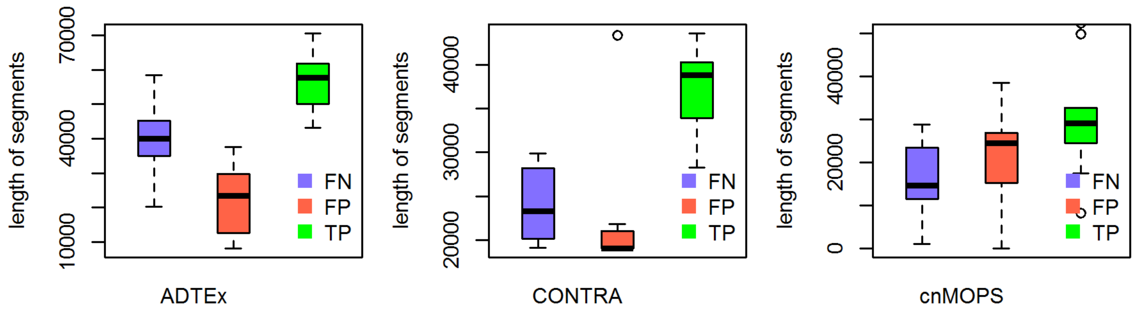

d) e)

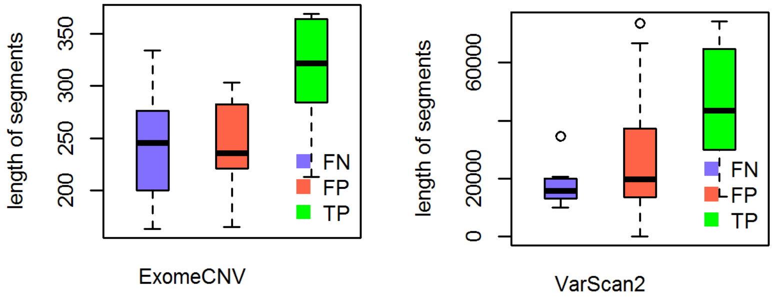

f)

TOOLS	P-Values
ADTEx	0.0036
CONTRA	0.0003
Cn.MOPS	0.0140
ExomeCNV	0.0311
VarScan2	0.0028

Fig. S2. The boxplots of lengths of FP, FN, and TP CNV segments for a) ADTEx, b) CONTRA, c) cn.MOPS, d) ExomeCNV, and e) VarScan2 for deletion . f) P-values from comparing lengths of FN and TP CNV segments using Wilcoxon test. (3M simulated data, Threshold=0.5)
